# COVID-19 and Dental and Dental Hygiene Students’ Career Plans

**DOI:** 10.1177/2380084420984772

**Published:** 2021-04

**Authors:** D.T. García, A.A. Akinkugbe, M. Mosavel, C.S. Smith, T.H. Brickhouse

**Affiliations:** 1 Department of Health Behavior and Policy, School of Medicine, Virginia Commonwealth University, Richmond, VA, USA; 2 Institute for Inclusion, Inquiry, and Innovation, Virginia Commonwealth University, Richmond, VA, USA; 3 Department of Dental Public Health and Policy, School of Dentistry, Virginia Commonwealth University, Richmond, VA, USA; 4 Department of General Practice, School of Dentistry, Virginia Commonwealth University, Richmond, VA, USA

**Keywords:** career choice, dental education, dental students, wellness, health workforce

## Abstract

**Objective:**

The aims of this study were to investigate whether dental and dental hygiene students’ career plans postgraduation were affected by the coronavirus disease 2019 (COVID-19) pandemic and to examine wellness and readiness for clinical practice among students who reported a change in career plans.

**Methods:**

An anonymous online REDCap survey was developed and emailed to 436 dental and dental hygiene students at a US dental school. The survey consisted of 81 questions that covered demographics, career plans postgraduation, and readiness and wellness measures. An open-ended question assessing how students’ career plans have changed during the pandemic was also included.

**Results:**

A total of 252 students completed the survey, of whom 11.5% reported that their plans for future dental practice have changed since the COVID-19 outbreak. Students who reported a change to their career plans had significantly higher mean perceived stress (20.1 vs. 16.3; *P* = 0.003) and anxiety (9.2 vs. 6.2; *P* = 0.004) scores and lower mean resilience (18.9 vs. 20.9; *P* = 0.01) scores than those who reported no change to their career plans. Concerns were raised regarding the limited employment opportunities, long-term stability of the dental profession, and the interruptions to clinical education and licensure examinations consequent to the pandemic.

**Conclusions:**

A comprehensive effort inclusive of adeptly designed clinical and curriculum experiences paired with wellness interventions and support tailored to students is needed. These measures need to support trainees across varying years in training and resilience levels to be effective for dental and dental hygiene students as they approach their future career intentions in the dental profession. Additional longitudinal research is needed to assess if change in career intentions during the COVID-19 pandemic corresponds with actual change postpandemic and affects the dental profession.

**Knowledge Transfer Statement:**

This study explores the potential short-term change in career intentions of dental hygiene and dental students during the COVID-19 pandemic. Findings can inform workforce planning as well as interventions developed and implemented by academic dental institutions to support student wellness during unexpected and prolonged emergency situations.

## Introduction

The coronavirus disease 2019 (COVID-19) pandemic has greatly affected health care delivery, particularly dental care (Health Policy Institute and American Dental Association 2020). Droplets and aerosols produced during most dental procedures represent possible routes of COVID-19 transmission, thus presenting concerns for patient and provider safety (Peng et al. 2020). In fact, the speculation and concerns related to the risk of COVID-19 infection within the dental environment led to the temporary disruption of clinical care at dental practices and prompted the development of guidelines by the Centers for Disease Control and Prevention (CDC) and the American Dental Association (ADA) to minimize the risk of transmission in dental care settings (ADA 2020).

This unprecedented pandemic has disrupted dental education and training. At the height of the pandemic, academic dental institutions responded by moving classroom instruction online and temporarily closing clinics (American Dental education Association 2020). A survey of student perceptions on dental education during the first wave of the pandemic in the United States revealed that these changes negatively affected preclinical and clinical training due to the lack of familiarity and fatigue of virtual learning and limitations related to hands-on practice and clinical experience (Van Doren et al. 2020). Disruptions to dental education and training also generated anxiety among students, who found themselves having to adjust to new educational instruction modalities while being fearful for their safety and health (Iyer et al. 2020). The new COVID-19–related stressors faced by students were in addition to other identified stressors traditionally associated with dental education such as academic workload and clinical requirements (Alzahem et al. 2011).

Although the impact of the pandemic on dental education and training is indisputable, its impact on students’ career trajectories is not well understood. Prior research on career motivations has found that dental hygiene students pursue their profession due to a desire to have a career in the health care field, serve the community, and receive good salaries (DeAngelis et al. 2003; Knevel et al. 2015). Similarly, dental students’ career motivation stems from their desire to help others, the prestige associated with the profession, and financial security (Avramova et al. 2014; Knevel et al. 2015). As students progress in their training, anticipated career plans may change based on a variety of factors such as educational debt, mentoring by faculty and staff members, and potential challenges securing admission into specialty programs or establishing a private dental practice (Nashleanas et al. 2014; Puryer and Patel 2016). The anticipated long-term impacts of COVID-19, such as reduced incomes and limited job opportunities, and the possibility of changing regulatory guidelines present additional external factors that may affect students’ career intentions (Barabari and Moharamzadeh 2020).

To date, the ramifications of the COVID-19 pandemic on students’ career intentions are not clear. Analyzing changes in students’ career plans is important in understanding the long-term effects of prolonged and unanticipated public health crises on the dental workforce. To this end, the aims of this study were to investigate whether dental and dental hygiene students’ career plans postgraduation were affected by the COVID-19 pandemic and to examine wellness and readiness for clinical practice among students who reported a change in career plans.

## Methods

An anonymous online REDCap survey (Harris et al. 2009) was developed and emailed to 436 dental and dental hygiene students enrolled at Virginia Commonwealth University School of Dentistry located in Richmond, Virginia, United States. Data collection occurred during a 3-wk period when the stay-at-home orders were in place in the Commonwealth of Virginia. At that time, the COVID-19 cases per 100,000 population 7-d average in the City of Richmond were on the rise, going from 4.2 at the start of the survey (April 27) to 14.2 at the end of the survey (May 17) (Virginia Department of Health 2020). There was also a temporary closure of the School of Dentistry and postponement of elective procedures during the survey period. Students were invited to participate in the study through an email that contained a unique link to the survey. Biweekly follow-up emails were sent to students to encourage participation. Interested students provided informed consent electronically prior to taking the survey. Individuals who completed the survey were compensated with a $10 online gift card. Prior to data collection, the study was reviewed and approved by Virginia Commonwealth University’s Institutional Review Board as exempt (IRB HM20019288).

The survey consisted of 81 questions that covered a wide range of topics, including demographics, anticipated educational debt, career plans postgraduation, and readiness to enter clinical practice or residency. The survey also incorporated previously validated scales with acceptable internal reliability to assess student wellness. These scales include the Perceived Stress Scale (PSS-10) (Cohen et al. 1983), Generalized Anxiety Disorder Scale (GAD-7) (Spitzer et al. 2006), Social Support Scale (OSS-3) (Kocalevent et al. 2018), Brief Resilience Scale (BRS) (Smith et al. 2008), and the Coping Scale (Hamby et al. 2013). These wellness measures have been previously described for this population (Akinkugbe et al. 2020).

This study focused on a subset sample from the broader survey consisting of students who responded yes to the question, “Since the COVID-19 outbreak, has your plans for future dental practice changed?” Descriptive statistics for demographics (sex, marital status, race and ethnicity, year in the dental program), anticipated educational debt, readiness to enter clinical practice/residency, and wellness scores (stress, anxiety, social support, resilience, and coping) by change in career plan (yes or no) were calculated using SAS 9.4 (SAS Institute). Differences in frequencies of responses to demographic and readiness measures were analyzed with Fisher’s exact test. Differences in mean wellness scores between survey participants who reported a change to their career plans and those who did not were assessed using an analysis of variance (ANOVA). *P* < 0.05 was deemed statistically significant.

Survey respondents who answered yes to a change in their future career plans since the COVID-19 outbreak provided responses to an open-ended question of “how their plans have changed.” Qualitative assessment of this open-ended question allowed the authors to expand beyond the close-ended question and explore the range of students’ responses to career plans change during the COVID-19 outbreak. Responses to the open-ended question were assessed using inductive thematic analysis conducted by 2 members of the research team (DTG, MM), with training in qualitative methods (Braun and Clarke 2006). Analysis of responses began using open coding and emergent categories, and resultant themes were discussed among authors. Discrepancies were resolved by discussion and reaching a consensus.

## Results

Of the 252 students who completed the online REDCap survey, 29 (11.5%) reported that their plans for future dental practice have changed since the COVID-19 outbreak (Table). More than half (58.6%) of students who reported a change in their career plans had anticipated going into private dental practice, while the majority of students who did not change their career plans reported an intention to pursue a residency/internship program postgraduation. About 40% of the students who reported a change in their career plans self-identified as Asian (41.4%) or were in their third year of dental school (41.4%) (Table). On the contrary, racially minoritized students were more likely than White students to report a change in their career plans. Specifically, 31% of Black and 19% of Latinx students as compared to 7% of White students reported a change to their career plans. When considering the entire survey sample (*n* = 252), a much higher proportion of fourth-year dental hygiene students (44.4%), followed by third- and fourth-year dental students (17.9% and 14.6%, respectively), reported a change in their postgraduation career plans. Slightly less females than males (10.6% vs. 12.6%, respectively) in the overall survey sample reported a change to their future career plans.

**Table.** Characteristics, Readiness, and Mean Wellness Scores of Survey Respondents by Reported Change in Career Plan Due to the COVID-19 Outbreak (*n* = 252).

Characteristic	Career Plan Changed (*n* = 29), *n* (%)	No Career Plan Change (*n* = 223), *n* (%)	*P* Value
Sex			0.7
Male	12 (41.4)	83 (37.2)	
Female	17 (58.6)	140 (62.8)	
Class year			0.001
D1	6 (20.7)	65 (29.2)	
D2	0	50 (22.4)	
D3	12 (41.4)	55 (24.7)	
D4	6 (20.7)	35 (15.7)	
DH3	1 (3.45)	13 (5.83)	
DH4	4 (13.8)	5 (2.24)	
Marital status			0.09
Single	17 (58.6)	156 (70.0)	
Married	10 (34.5)	64 (28.7)	
Other	2 (6.90)	3 (1.35)	
Race/ethnicity			0.09
White	9 (31.0)	115 (51.6)	
Black	4 (13.8)	9 (4.04)	
Asian	12 (41.4)	75 (33.6)	
Latinx	4 (13.8)	17 (7.62)	
American Indian/Alaskan Native/Native Hawaiian/ Pacific Islander	0	1 (0.45)	
Two or more races	0	6 (2.69)	
Anticipated educational debt			0.04
No debt to <$50,000	10 (34.5)	59 (26.5)	
$50,001 to $200,000	4 (13.8)	92 (41.3)	
$200,001 to $350,000	9 (31.0)	42 (18.8)	
>$350,000	6 (20.7)	30 (13.5)	
Career plan postgraduation			0.03
Private dental practice	17 (58.6)	74 (33.2)	
Residency/internship	5 (17.2)	94 (42.2)	
Other^a^	4 (13.8)	24 (10.8)	
Unsure	3 (10.3)	31(13.9)	
Confidence entering clinical practice/residency			0.4
Very or somewhat confident	22 (75.9)	152 (68.2)	
Not too or not confident at all	7 (24.1)	71 (31.8)	
	Mean (SD)	Mean (SD)	*P* Value
Generalized Anxiety Disorder Scale (GAD-7)	9.2 (5.9)	6.2 (5.1)	0.004
Perceived Stress Scale (PSS-10)	20.1 (5.2)	16.3 (6.6)	0.003
Social Support Scale (OSS-3)	10.3 (2.5)	11.1 (2.1)	0.08
Brief Resilience Scale (BRS)	18.9 (3.4)	20.9 (4.3)	0.01
Coping Scale	38.8 (7.0)	39.1 (5.8)	0.70

a Other includes federally qualified health center, academia, dental service organization/corporate dental practice, armed forces, and other nonprofit clinic.

Comparison of readiness to enter the clinical practice/residency and wellness measures between students who reported a change and those who did not report a change in their future career plans due to the COVID-19 outbreak revealed noteworthy differences. For instance, the 2 groups differed significantly on anxiety (9.2 vs. 6.2; *P* = 0.004), stress (20.1 vs. 16.3; *P* = 0.003), and resilience scores (18.9 vs. 20.9; *P* = 0.01). There was no significant difference between groups with respect to social support (10.3 vs. 11.1; *P* = 0.08), coping (38.8 vs. 39.1; *P* = 0.70), or self-reported readiness to enter clinical practice or residency (*P* = 0.4).

Qualitative analysis of the open-ended question, “Since the COVID-19 outbreak, has your plans for future dental practice changed? If yes, how?” yielded 3 themes: employment concerns, disruptions, and uncertainty; pursuing alternative career plans; and concerns about the stability of the dental profession.

### Employment Concerns, Disruptions, and Uncertainty

Among final-year dental and dental hygiene students, 40% reported that they have not been able to secure their desired postgraduation employment. Some students reported difficulties finding any employment during the COVID-19 pandemic. Others were unable to secure a position in a location they desired or stated that they anticipate working part-time rather than full-time upon graduation. The main reported barrier to obtaining their desired employment during the COVID-19 pandemic was the limited opportunity to communicate with practice owners due to closures resulting from the stay-at-home orders. Half of the fourth-year dental hygiene students also identified interruptions to licensure examination as a major barrier to postgraduation employment. Indeed, a fourth-year dental hygiene student indicated a lack of willingness to pursue an initial career plan with the Indian Health Service (IHS) due to delays in obtaining licensure, as noted here:

My original plan was to work with IHS as a public servant but I need my license to apply. I cannot apply for it until August now due to all exams being pushed back. Therefore, I will miss the application deadline for the program I wanted to work for. (Female, DH4 student)

The COVID-19 pandemic also influenced third-year and nonclinical-year dental students’ perceptions of the future job market and their readiness to enter it. Several students reported feeling unsure of the imminent job environment; as one student stated, “We do not know how long this outbreak will last.” Students’ perception of a forthcoming dismal job market stemmed from concerns regarding the long-term financial strain of COVID-19 on dental schools and/or dental offices. Specifically, students reported uncertainty on whether or not dentists will be hiring associates in their private practice consequent to the reduced patient volumes attributed to the COVID-19 pandemic. Third-year dental students also reported feeling less confident in applying to residency programs or entering private practice directly given the extended interruption to their clinical education. For 1 third-year dental student, the academic disruption to his clinical education left him feeling anxious about his ability to accomplish certain clinical procedures prior to applying to residency programs, as noted here:

Anxiety about [my] ability to accomplish certain procedures during limited time in dental school that may limit my ability to be a qualified candidate while applying to residencies. (Male, D3 student)

### Pursuing Alternative Career Plans

Given the heightened sense of uncertainty regarding the impact of the ongoing COVID-19 pandemic on the dental job market, several students reported they will pursue alternative career plans postgraduation. Among dental hygiene students, 1 student reported they will enroll in a master’s of public health program instead of joining the dental workforce as initially planned. Similarly, the most common alternative career plan mentioned by dental students across all years of training was enrolling in a residency program. However, students pursuing this option reported facing additional barriers such as having to take on additional student debt as well as not being able to travel and visit such programs in person before applying due to travel restrictions brought on by the pandemic.

I am very concerned that dental schools and/or dental offices will not be hiring due to the financial strain of COVID-19. I may not be able to find employment and may need to find a residency program to continue to practice dentistry, which will require me to take on more student loan debt. (Female, D3 student)

Similarly, first-year dental students also reported change to their future career plans due to the COVID-19 pandemic. One student who initially planned to specialize in oral and maxillofacial surgery (OMFS) reported she will no longer pursue that specialty after learning of a COVID-19–related death of an OMFS resident. Instead, she is now considering completing 1 or 2 y of residency or joining the dental workforce sooner.

Initially I planned on specializing but since the COVID-19 outbreak and learning about the death of the OMFS resident in Detroit, I much rather do one or two years of GPR or AEGD and join the workforce sooner. (Female, D1 student)

### Concerns about the stability of the dental profession

For several students, the COVID-19 pandemic influenced their perception of the overall stability of the dental profession. The closures of dental practices due to the outbreak left some students questioning the long-term job security of their chosen profession. Students shared statements such as “I thought dentists had some of the best job security” and “I don’t know if this is what makes me happy anymore—no security, lack of communication.” Some students stressed needing to be prepared in case another outbreak occurs while others were unsure if they should be doing more to prepare themselves, as noted here:

I thought Dentists had some of the best job security and didn’t foresee any reason dentists would have to close their practices like this for an extended period of time. Not knowing what the future “normal” will look like when the COVID-19 outbreak settles down makes me slightly anxious about the future and has made me question whether my plan for the future is as secure as I thought it was or if I need to do more. (Female, D1 student)

Other students expressed concern over the potential long-term impact of the pandemic on the dental profession and how it is practiced. One student was particularly concerned that the reduced patient volume would negatively affect his ability to pay off dental school loans and purchase his own private practice in the future. Furthermore, this student also expressed concern over the possible shift toward teledentistry as well as ongoing health risks for patients, staff, and family, as noted here:

The average debt amount we will be graduating in 2023 will be $500K at 7% interest rate, which is $35,000 of accrued annual interest alone. Paying this loan aggressively only makes sense if your income is $250K+ after taxes, but realistically it will take over a decade to do so, and making this move severely limits loan borrowing power when looking to purchase a private practice. 20 year IDR plans like REPAYE are much better options. The biggest concern is patient volume, increased cost of care, reduced hiring for associate dentists, permanent teledentistry shift (don’t like it, lack of control), health concerns for my patients, staff, and my family. (Male, D1 student)

## Discussion

The purpose of this study was to investigate whether the postgraduation career plans of dental and dental hygiene students at a US dental school were affected by the COVID-19 pandemic. To our knowledge, this is the first study to examine the short-term impact of this pandemic on dental and dental hygiene students’ career intentions. Furthermore, findings from this survey, which was administered from April 28 to May 17, 2020, provide a baseline understanding of COVID-19–related changes in career intentions within this population to allow for future assessment as the pandemic continues to evolve.

Over 10% (11.5%) of survey respondents reported a change to their future career plans due to the COVID-19 pandemic, with the highest proportion of change reported among fourth-year dental hygiene students and clinical-year dental students (D3 and D4). These students expressed COVID-19–related employment concerns stemming from interruptions to licensure examinations and their inability to communicate with practice owners during practice closures. This is not surprising to find given that at the time of the survey, only 3% of dental practices in the United States were open with business as usual (Health Policy Institute and American Dental Association 2020). This finding is also consistent with prior studies that found that graduating dental students are most concerned about their professional future and career prospects (Polychronopoulou and Divaris 2005) and that more senior dental hygiene students do not associate the profession with job security or diverse career opportunities as highly as compared to first-year students (DeAngelis et al. 2003). Interestingly, we also found that slightly more male than female survey participants reported a change to their future career plans during the pandemic, suggesting that the influence of broader cultural norms and social forces may be at play. For example, within the dental field, there is a persistent gender gap in earnings, which is believed to be due in part to the fact that women are more likely to treat Medicaid patients and focus on preventive procedures, both of which are reimbursed at lower rates (Vujicic et al. 2017). As such, the slightly lower proportion of female than male participants who reported a career change due to the pandemic may actually be reflective of the broader inequitable expectation that women will earn less than men regardless of the long-term financial strain that COVID-19 has on the field. This is an area of research that should be further explored in future studies.

Nonetheless, our findings suggest that dental and dental hygiene students who reported a change in career plans actually experienced a career shock, defined as disruptive and extraordinary events, such as the COVID-19 pandemic, that trigger a deliberate thought process concerning one’s career (Akkermans et al. 2018). We found that study participants as early as their first year of dental training experienced a COVID-19 career shock that triggered a change in career plans, underscoring the profound impact that the pandemic has had on students’ career aspirations. In particular, students across all years of training expressed concern regarding the future stability of the dental profession due to the pandemic. Concerns regarding the looming dismal job market prompted several students to consider a change to their career plans and instead pursue residency training despite having to accrue more educational debt in the process. These fears and anxiety were also visible among dental practitioners who had to modify their services and temporarily close their practices at the peak of the pandemic (Ahmed et al. 2020). However, a lot has changed in the months since this survey was conducted, including the publication of a study showing low COVID-19 prevalence and testing positivity rates among licensed US dentists (Estrich et al. 2020). Another study conducted over a 6-mo period in 3 dental offices in New York found that using screening questionnaires, following infection control guidelines, and using personal protective equipment led to no transmission of COVID-19 to dental health workers or patients during the study period (Froum and Froum 2020). Findings from these studies paint a more promising picture for the dental field moving forward and consequently also for students’ future career plans. Literature on career shocks indicates that the impact that disruptive events have on people’s careers depends on the event’s frequency, controllability, intensity, valence, and duration (Akkermans et al. 2018). Given the evolution of the ongoing pandemic and its impact on the dental field, future studies should conduct a longitudinal assessment to examine how student career intentions continue to evolve throughout the pandemic and beyond.

Findings from this study also revealed that racially minoritized students were more likely than White students to report a change in their career plans due to the COVID-19 pandemic. This finding may be reflective of the underlying inequities in education and the broader sociopolitical context that minoritized students experience, including the pervasive prominence of racism compounded with the disproportionate burden of the pandemic on minoritized communities (Bailey and Moon 2020). A potential decrease in the workforce of underrepresented dental providers may have long-term implications not just for dental training programs that have historically benefited from applicant pools to fill available training spots but also in the delivery of dental services to underserved communities. Underrepresented dental providers are more likely than White dental providers to work in racial and ethnic minoritized communities (Mertz et al. 2016). These dental providers also treat a disproportionate number of racially minoritized patients as well as patients covered by public insurance. Further assessment of how the COVID-19 pandemic continues to affect the career intentions of racially minoritized dental and dental hygiene students is warranted.

Examination of wellness measures showed that students who reported a change in their career plans due to the COVID-19 pandemic had significantly higher anxiety and perceived stress scores compared to those who did not report a change in future career plans. Similarly, a study conducted with licensed US dentists at the height of the pandemic found that 19.5% of participants reported symptoms of anxiety, underscoring the psychosocial impact of the pandemic across students and providers in the dental field (Estrich et al. 2020). Students who reported a change in their career plans also had lower resilience scores compared to those who did not report a change in their plans, corroborating prior evidence that resilience is a key psychological resource that could make career shocks more manageable (Akkermans et al. 2020). Resilient individuals show career success, despite adversities, due to their strong ability to plan and organize, often denoted as “planfulness” (Graner et al. 2018). Given these findings, academic dental institutions need to prioritize the creation of responsive academic environments that not only best prepare students for their future careers but also address concerns related to their well-being. The development and refinement of student wellness programs and even student support services offered to dental students could benefit from the greater understanding of how students matriculate through academically rigorous dental curriculums while traversing major life events such as the COVID-19 pandemic (Smith et al. 2020).

While current dental school curriculums leave little room for additional contact hours, the overwhelming association of resilience as a protective factor for mental health disorders and stress, as well as documented evidence of higher trending burnout in clinical practitioners (Calvo et al. 2017), lends itself to the necessity of incorporating resilience and wellness practices into dental and dental hygiene training programs. Career change as a result of COVID-19, as influenced by stress and lower resilience, contributes to the existing body of research that dental schools ought to integrate stress management programs into their curricula (Bhat and Basson 2013) or partner more collaboratively with university counseling services (Laurence et al. 2009). Counseling services embedded within dental schools themselves can help an organization better understand the needs of students and the utility of comprehensive services, as well as create an environment more sensitive and receptive to dental students’ mental health needs (Adams 2017). Integration of such practices as mindfulness, mediation, and other wellness modalities has been encouraged in both dental and medical education (Lovas et al. 2008). Examples include simple lectures, 1-d workshops, and even 8- to 10-wk program modules in mindfulness-based stress reduction (Dobkin and Hutchinson 2013). In addition to mindfulness practice, future studies could investigate the potential influence of disaster preparedness, crisis leadership, and change management on dental and dental hygiene student career change and mental health.

### Strengths and limitations of study

As with all studies, there are limitations to this work. First, findings represent students’ self-reported intended change in career plan due to the COVID-19 pandemic and not necessarily the actual future career path. Career plans might continue to change, positively and negatively, as the COVID-19 pandemic continues to evolve (Akkermans et al. 2020). Second, the survey instrument was 81 questions long, which may have contributed to participant fatigue and decreased reporting. Third, the study sample represents the views of students at 1 US dental school, which limits the generalizability of study findings. Longitudinal assessment of how career intentions change throughout the duration of the COVID-19 pandemic among students across US dental schools is warranted. Last, the qualitative assessment of how career plans changed during the COVID-19 pandemic was based on responses to an open-ended survey question and not from a formally structured interview guide based on qualitative inquiry. However, the judicious use of open-ended questions is a methodological approach that provides valuable insights into the meaning and quality of information provided by survey respondents (Singer and Couper 2017). Future studies should use the strength of qualitative methods to examine the impact of the COVID-19 pandemic on dental and dental hygiene students’ career intentions.

## Conclusion

This study examined the short-term impact of the COVID-19 pandemic on dental hygiene and dental students’ career intentions. Findings demonstrate that students who reported an intent to change their career plans had significantly higher perceived stress and anxiety scores and lower resilience scores than students who reported no change to their career plans. Racially minoritized students were more likely to report a change in career plans than White students were. Students who reported a change in their career plans were concerned about limited employment opportunities, the long-term stability of the dental profession, and interruptions to clinical education and licensure examinations consequent from the pandemic. It is important that dental training programs have the financial and institutional support to provide safe and supportive in-person, virtual, and clinical training environments with the communication structures to support students and build a confident and resilient dental workforce.

## Author Contributions

D.T. García, contributed to conception, design, data acquisition, analysis, and interpretation, drafted and critically revised the manuscript; A.A. Akinkugbe, contributed to conception, design, data acquisition, analysis, and interpretation, critically revised the manuscript; M. Mosavel, contributed to conception, design, data analysis, and interpretation, critically revised the manuscript; C.S. Smith, T.H. Brickhouse, contributed to conception and design, critically revised the manuscript. All authors gave final approval and agree to be accountable for all aspects of the work.
